# Ameliorative effect of vanillin on scopolamine-induced dementia-like cognitive impairment in a mouse model

**DOI:** 10.3389/fnins.2022.1005972

**Published:** 2022-11-04

**Authors:** Abhinav Anand, Navneet Khurana, Nemat Ali, Abdullah F. AlAsmari, Metab Alharbi, Mohammad Waseem, Neha Sharma

**Affiliations:** ^1^School of Pharmaceutical Sciences, Lovely Professional University, Phagwara, Punjab, India; ^2^Department of Pharmacology and Toxicology, College of Pharmacy, King Saud University, Riyadh, Saudi Arabia; ^3^School of Pharmacy, University of Maryland Eastern Shore, Princess Anne, MD, United States

**Keywords:** acetylcholinesterase, Alzheimer’s disease, antioxidant, dementia, scopolamine, vanillin

## Abstract

**Background:**

Alzheimer’s disease (AD) is the most common form of dementia, which is among the top five causes of death in the United States. It is a neurodegenerative disorder that causes permanent loss of memory and cognition. The current pharmacotherapy for AD is based on providing symptomatic relief only and has many side effects. There is a need for a safer, disease-modifying drug for the treatment of AD.

**Experimental approach:**

The PASS online software was used to screen phytoconstituents based on their predicted effects on various AD-related targets. Vanillin was selected as the compound of interest, as it has not been researched elaborately on any animal model of AD. The acetylcholinesterase inhibitory activity of vanillin was established *in vitro*. Thereafter, ameliorative effect of vanillin was evaluated using the exteroceptive memory model in scopolamine-induced cognitive impairment mice model.

**Results:**

Vanillin showed an acetylcholinesterase inhibitory activity *in vitro*, and the IC_50_ value was calculated to be 0.033 mM. Vanillin significantly reversed the memory and behavioral deficits caused by scopolamine as demonstrated by significant improvement in memory in negative reinforcement, elevated plus maze, and spatial learning paradigms. Vanillin also proved to have a nootropic effect. Also, vanillin proved to have significantly better antioxidant and acetylcholinesterase inhibitory effects *in vivo* than donepezil hydrochloride. The potential anti-AD activity of vanillin was also confirmed by the reduction in IL-6 levels and TNF-α levels.

**Conclusion:**

Our results suggest that vanillin is a safe and effective natural drug candidate having a great potential for the treatment of AD. However, more research is required to evaluate its effect on A beta plaques and Tau neurofibrillary tangles *in vivo*.

## Introduction

Dementia is more of a progressive disorder manifesting impairment in memory and cognitive abilities than a disease. Neurodegenerative dementia is age-dependent and has an estimated prevalence rate of 20% in geriatric individuals (more than 80 years of age). Alzheimer’s disease (AD) is the most common form of dementia (Anand et al., 2017a; Sharma et al., 2017). AD incorporates events ranging from early memory lapses to patient being functionally dependent. Ultimately, death is inevitable (Neugroschl and Wang, 2011). The pathological hallmarks of AD include deposition of amyloid beta (Aβ) plaques and tau neurofibrillary tangles (Nisbet et al., 2015; Morgese et al., 2017), decrease in brain acetylcholine (ACh), overexpression of acetylcholinesterase (AChE), and glutamate excitotoxicity (Francis, 2005). Oxidative stress plays a major role in the development of AD because of its neurodegenerative impact (Huang et al., 2016). The current pharmacotherapy for AD involves the usage of acetylcholinesterase (donepezil, rivastigmine, and galantamine) and NMDA glutamate (memantine) inhibitors, both of which only provide symptomatic relief along with several side effects (Anand et al., 2017b). There is a need for a safer, more effective, and disease-modifying drug for the treatment of AD (Geerts et al., 2017; Musardo and Marcello, 2017).

PASS online is a type of software that predicts the biological activity of compounds based on 2D structures and canonical SMILES (Marwaha et al., 2007). In the present study, it has been used to screen the compound of interest, i.e., vanillin for its predicted effects on AD-related targets. In previous studies, vanillin has been reported to have an Aβ disintegratory effect *in vitro* (Song et al., 2016) and an antioxidant effect *in vivo* (Dhanalakshmi et al., 2016).

In the present study, an *in vivo* evaluation of vanillin has been conducted on a scopolamine-induced dementia model of mice for its neuroprotective, antioxidant, and ameliorative effects. Scopolamine is a parasympatholytic agent that has been reported to produce amnesia in humans along with causing impairment of cognitive processes in animals. It alters the activity of multiple genes that play a role in cholinergic signaling pathways mediated by muscarinic receptors, apoptotic cell death of neurons, and cell differentiation in the brain. Cortical parasympathetic neurons are subjected to malfunction and degeneration, which is not different from the cognitive impairment that is seen in the AD type of dementia (Lieben et al., 2005). Administration of scopolamine causes elevation in the levels of reactive oxygen species, consequently leading to oxidative stress. Scopolamine has also been reported to cause behavioral deficits (Goverdhan et al., 2012; Abd-El-Fattah et al., 2014). Chronic administration of scopolamine can also induce amyloid beta deposition, synaptic dysfunction, and learning/memory impairment, as seen in AD (Chen et al., 2014). Therefore, prolonged administration of scopolamine in animals is commonly used to develop an animal model of AD that mimics general human dementia, specifically for AD (Ogura et al., 2000; Blokland et al., 2006; Kwon et al., 2009).

Based on previously reported endpoints in the literature, inhibitory activity is a measure of the memory-enhancing potential of acetylcholinesterase (Tabet, 2006). Also, assessment of escape latency (Bromley-Brits et al., 2011), step-down latency, and transfer latency (Habibyar et al., 2016) serves as a measure of enhancement in memory and cognition. Evaluation of various biochemical parameters like TBARS, GSH, and catalase activity is the measure of antioxidant and therefore neuroprotective activities of the compound (Habibyar et al., 2016). IL-6 (Lyra e Silva et al., 2021) and TNF-α (Chang et al., 2017) have been reported to play a pivotal role in the pathogenesis and progression of neurodegenerative disorders, such as AD. Therefore, in the present study, the ameliorative effect of vanillin on scopolamine-induced dementia has been evaluated in the aforesaid parameters.

## Materials and methods

### PASS online program

Vanillin has been shown to exhibit neuroprotective and other associated activities, which strongly rationalize its evaluation for anti-dementia effect. Furthermore, the PASS program has been employed to affirm the rationale. PASS is an online type of software that predicts the pharmacological activities of various compounds. Numerous studies have been reported that employed the assistance of the PASS program for predicting the biological activities of chemical substances (Marwaha et al., 2007; Goel et al., 2010; Khurana et al., 2011). The probable activity of vanillin for its effect on dementia and parameters related to oxidative stress was analyzed using this program (Anand et al., 2017c). After the aforementioned activities were predicted, the *in vivo* study was carried out using a scopolamine-induced dementia-like cognitive impairment model of mice.

### Evaluation of acetylcholinesterase activity of vanillin (*in vitro*)

Acetylcholinesterase is an enzyme that is responsible for the hydrolysis of acetylcholine. As established by the cholinergic hypothesis of AD, decreased level of ACh in the brain is responsible for the decline in cognition and memory (McGleenon et al., 1999; Francis, 2005; Garcia-Alloza et al., 2005). Therefore, acetylcholinesterase inhibitory activity is a desirable characteristic for a compound to be taken as a potential drug candidate for the treatment of AD.

The acetylcholinesterase activity of vanillin was evaluated using modified Ellman’s method (Ellman et al., 1961; Atta-ur-Rahman and Choudhary, 2001; Hasnat et al., 2013).

To 1,500 μL of Sorenson’s phosphate buffer (pH 8), 100 μl of a vanillin solution was added. To this solution, 200 μl of an acetylcholinesterase enzyme (from electric eel) solution (0.1 units/ml) was added, and the solution was incubated at 25°C for 15 min. Then, 100 μl of Ellman’s reagent, i.e., DTNB (10 mM) was added to the above solution. The reaction was started by adding a substrate, i.e., 100 μl of acetylcholine bromide (14 mM) solution. The hydrolysis of the substrate was measured by the formation of the 5-thio-2-nitobenzoate anion (a colored product formed when the thiocholine released from ACh reacts with DTNB). The absorbance of the sample was measured at 410 nm after 10 min using a double-beam UV spectrophotometer (Shimadzu). Donepezil at a final concentration of 10 μM in the assay was employed as a positive control, and the same process was followed. The inhibitory activity of vanillin was calculated by using the formula:


P⁢e⁢r⁢c⁢e⁢n⁢t⁢a⁢g⁢e⁢i⁢n⁢h⁢i⁢b⁢i⁢t⁢i⁢o⁢n=

(1-a⁢b⁢s⁢o⁢r⁢b⁢a⁢n⁢c⁢e⁢o⁢f⁢s⁢a⁢m⁢p⁢l⁢e/a⁢b⁢s⁢o⁢r⁢b⁢a⁢n⁢c⁢e⁢o⁢f⁢c⁢o⁢n⁢t⁢r⁢o⁢l)×100.

The assay was repeated for various concentrations of vanillin. All the concentrations were assayed in triplicate.

The concentration of vanillin that inhibited the hydrolysis of acetylcholine by 50% (IC_50_) was determined by linear regression analysis between percentage inhibition and the concentration in the Microsoft Excel program (Niño et al., 2006; Saleem Ali-Shtayeh et al., 2014). Assay solutions were made at concentrations in the range 10^–3^–10^–5^ M (Hamulakova et al., 2017).

### Animals

Adult Swiss albino mice (either sex; 20–30 g each) were procured from the National Institute of Pharmaceutical Education and Research (NIPER), S.A.S. Nagar, Punjab (a CPCSEA-registered breeding facility). The animals were transported by road using the institutional van to avoid transport stress. Polypropylene cages of appropriate size were provided to enable the animals to have comfortable, free movements and protection from possible injuries. Food and water were provided in suitable containers and appropriate forms to ensure that they get adequate food and more particularly water during transit. They were kept in the central animal house facility at Lovely Institute of Technology (Pharmacy), Lovely Professional University (Phagwara, Punjab) registered with the Committee for the Purpose of Control and Supervision on Experiments on Animals (CPCSEA). The animals were given 12-h light and 12-h dark cycles and were kept under ambient temperature and humidity conditions. Food and water were given *ad libitum*. All the procedures were conducted as per the guidelines of CPCSEA. The protocol for the use of animals for this study was approved by the Institutional Animal Ethics Committee, Lovely Professional University (Phagwara, Punjab).

### Drugs and chemicals

Scopolamine hydrobromide trihydrate and the acetylcholinesterase enzyme (from electric eel) were purchased from Sigma-Aldrich (now Merck). Donepezil hydrochloride monohydrate was received *ex gratis* from Wockhardt Research Center, Aurangabad, Maharashtra (India). Vanillin was purchased from LobaChemie Pvt. Ltd., Mumbai, Maharashtra (India).

### Experimental design

#### Exteroceptive memory model

The animals were assigned to four different groups (*n* = 5/6). Group 1 served as a control group that received the vehicle (normal saline) as a drug at a volume of 5 ml/kg through the p.o. route for 10 consecutive days. Group 2 served as the standard treatment group (Dpz *per se*) and was administered donepezil hydrochloride (5 mg/kg) in normal saline (5 ml/kg p.o.) for 10 consecutive days. Groups 3 and 4 served as test drug treatment groups (V10 and V20 *per se*) and were administered vanillin (10 mg and 20 mg/kg p.o., respectively, in 5 ml/kg normal saline) for 10 consecutive days (Dhanalakshmi et al., 2016; Habibyar et al., 2016).

#### Scopolamine-induced dementia model

All the mice were assigned to five groups (*n* = 5/6). Group 1 served as the vehicle control and received 5 ml/kg normal saline (p.o. and i.p.). Group 2 (Sco *per se*) was the negative control/disease control that was administered scopolamine hydrobromide trihydrate 2 mg/kg (i.p.). Group 3 (Dpz + Sco) served as the standard treatment group that received the standard drug, i.e., donepezil hydrochloride monohydrate 5 mg/kg (p.o.) and scopolamine hydrobromide trihydrate 2 mg/kg (i.p.). Groups 4 and 5 (V_10_ + Sco and V_20_ + Sco) received vanillin (10 and 20 mg/kg, p.o. respectively) along with scopolamine hydrobromide trihydrate 2 mg/kg (i.p.). All of the mentioned treatments were conducted for 10 consecutive days (Goverdhan et al., 2012; Dhanalakshmi et al., 2016; Habibyar et al., 2016). Sterile normal saline was used to prepare all the drug solutions under aseptic conditions.

### Evaluation of locomotor activity

For the evaluation of spontaneous locomotion in the test mice, locomotor activity was assessed. Each animal was subject to the test in an actophotometer (Inco, India) individually. The instrument comprised a square activity chamber. For facilitating the placement and removal of the mice, the chamber had a removable opaque lid. There were 12 holes close to the bottom of the activity chamber. Photoelectric emitters and receivers were attached to the holes. The sensors are arranged in a manner such that any movement of a mouse will block the light beam’s path associated with at least one sensor, and gets registered and is displayed on the digital activity meter.

Each mouse was individually placed in the activity chamber for 5 min (after half an hour of i.p. treatment and/or 1 h of oral treatment). The locomotor activity of the animals was recorded on the 0th, 2nd, 5th, and 9th days of the treatment (Khurana et al., 2011; Nazari et al., 2013; Habibyar et al., 2016).

### Evaluation for ameliorative effect on the basis of behavioral parameters

The memory of the animals was evaluated by the step-down latency (SDL) test in passive avoidance paradigm, the transfer latency (TL) test in elevated plus maze paradigm (Vasudevan and Parle, 2008; Khurana et al., 2011), and the escape latency (EL) test in Morris Water Maze paradigm (Bromley-Brits et al., 2011).

#### Transfer latency test

The TL test was conducted using an “elevated plus maze” as a behavioral model for evaluation of memory and cognition in mice. The protocol, method, and end point for the test were based on the parameters given in some previous investigations (Parle et al., 2004; Dhingra and Kumar, 2012). The apparatus consisted of two closed arms (16 cm × 5 cm × 15 cm) and two open arms (16 cm × 5 cm) extending from a central platform (5 cm × 5 cm). The maze was placed at an elevation of 25 cm from the ground. During the acquisition trial, each mouse was gently kept at the edge of one open arm, facing away from the central platform. TL was defined as the time taken by the animals to enter (with all its four paws) in either of the closed arms from the open arm. For acquisition, TL was recorded for all the animals on the 10th day of the protocol individually. If an animal failed to enter into either of the closed arms within the cutoff time of 90 s, it was gently guided with hands into either of the two closed arms and TL was recorded as 90 s. The mice were left to explore the maze for 2 min before returning them to the home cage. Memory, as indicated by the retention of this learned task, was evaluated on the 11th day of the protocol, i.e., 24 h after the acquisition trial was concluded.

##### Parameters

Significant decrease in TL value during the retention tests indicates improvement in memory. However, it should be noted that if the drug under evaluation has a sedative effect or leads to muscular incoordination, then it may cause interference in the proper evaluation of retention. TL will appear to be decreased post-drug administration during the retention phase of the evaluation; therefore the drug may show false negative results.

#### Step-down latency test

Negative reinforcement-based passive avoidance was used to evaluate the memory of the mice (Kameyama et al., 1986; Sharma and Kulkarni, 1990; Khurana et al., 2011; Habibyar et al., 2016). The step-down paradigm apparatus comprised of a box (27 cm × 27 cm × 27 cm) having three walls of wood and one of Plexiglass. It contained a grid floor which is made up of 3-mm stainless steel rods set 8 mm apart. It consist of an insulated platform, made of thick plastic sheet (10 cm × 7 cm × 1.8 cm) in the center of the grid floor. The apparatus was illuminated with a 15-W bulb during the experimental period. Electrical shock was delivered *via* the grid floor.

In the training test on the 10th day of the protocol, each mouse was placed gently on the plastic platform kept in the middle of the grid. As soon as it descended with all its four paws, a foot shock was delivered for a duration of 1 s (50 Hz, 1.5 mA), and the mouse was immediately returned to the home cage. The step-down latency (SDL) was recorded as the time taken by the animals to step down from the plastic platform to the grid floor with all its four paws. A retention test was performed on the 11th day of the protocol. The mouse was placed on the platform as before and the SDL was recorded. The cut-off time for the SDL test was kept at 60 s.

##### Parameters

The SDL during acquisition and retention trials was recorded. Prolongation of SDL suggests improvement in memory. However, it should be noted that if the drug under evaluation has a sedative effect or leads to muscular incoordination, then it may cause interference in the accurate evaluation of retention. SDL may appear to be increased post-drug treatment during the retention phase of the evaluation; therefore, there may be a possibility of obtaining false positive results.

#### Escape latency test

The Morris Water Maze (MWM) was established by Morris (1981) a neuroscientist, in for evaluation of hippocampus-dependent learning (including acquisition of spatial memory and retention of the same) (Morris, 1981). The MWM is a relatively simple paradigm generally comprising trials running for a period of 6 days. The merits of this procedure include efficient differentiation between “non-spatial” (visible platform) and “spatial” (hidden platform) conditions and reduction in interference due to odor trail (Scoville and Milner, 1957; O’Keefe, 1979; Eichenbaum et al., 1990; Block, 1999). The MWM plays a pivotal role while validating rodent models for “neurocognitive disorders” like AD (Bromley-Brits et al., 2011).

The tendency of rodents to escape from water is relatively independent of variations in activity or body mass, thereby making it apt for several experimental models (Vorhees and Williams, 2006). The modified protocol by Bromley-Brits et al. (2011) was used as described below:

##### Pre-experimental preparation

A circular pool was taken with a diameter of 150 cm and depth of 50 cm. Because albino mice were used, the pool was colored black from the inside. The arrangement was made using plastic sheet dividers so the animals could not see the experimenter during the trials. Some “high-contrast spatial cues” were placed in the room and the inside of the pool above the surface where water would be. A 10 cm × 10 cm platform was placed in the pool. The pool was filled with water until the surface of water was just 1 cm below the surface of platform. The water was allowed to equilibrate to 22°C. The temperature should be kept constant throughout the protocol. The pool was divided into 4 quadrants.

##### Day 6 (Training trial)

The mice were brought from the housing area to the experimental room. The cages were kept in an area where the mice could not see the pool or the spatial cues. A period of 30 min was allowed for acclimatization. A flag was placed on the platform to make it more visible.

A mouse to be tested was lifted by the base of its tail and supported on a sponge as it was being brought to the testing area. The mouse was gently placed in the water facing the edge of the pool by lifting it up by the base of its tail. The testing area was immediately evacuated, and observations were made from a place invisible to the mouse.

If the mouse found the platform before the cutoff time of 60 s, it was allowed to remain on the platform for 5 s before being returned to its home cage. If the mouse could not find the platform within 60 s, it was directed toward the platform and was allowed to remain on it for 20 s before being returned to its home cage. The procedure was repeated for all the mice in the trial. Each mouse was subjected to 5 trials in a day with an inter-trial difference of 5 min. Each trial was initiated from a different starting direction. The EL, i.e., the time taken by each mouse to climb the platform, was recorded. After the testing was completed, the mice were dried off, and normothermia was ensured before returning them to the housing area.

##### Days 7–10 (Acquisition trials)

The water was made opaque by adding a titanium dioxide suspension, and more water was added so that the platform is submerged 1 cm below the surface of the water. The flag was removed from the platform. The procedure was repeated as above for all the mice on all days.

##### Day 11: Probe trial (Retention)

The platform was removed from the pool. The starting direction was kept same for all the trials, i.e., the direction farthest from the platform quadrant on previous days. The same procedure as above was repeated. The time of entry into the target quadrant (the quadrant that previously housed the platform), time spent in the target quadrant, and number of crossings near the position of the platform on previous days were recorded.

##### Parameters

A decrease in EL is regarded as an improvement in learning. A higher percentage of time spent in the target quadrant during the probe trial is interpreted as a higher level of memory retention.

### Biochemical estimation

With the conclusion of behavioral studies on the last day of the protocol, the animals were sacrificed. For assessment of biochemical parameters, the animals were euthanized by decapitation after cervical dislocation. The brains were immediately removed, and the cortical and hippocampal regions were separated on ice. The weight of the isolated parts was recorded and, in turn, the tissues were homogenized (10% w/v homogenate) using 0.1 M phosphate-buffered saline (PBS, pH = 7.4). Then, the mixture was centrifuged in a cooling centrifuge at 10,000 *g* (at 4°C for 15 min) to remove unnecessary cellular components (Habibyar et al., 2016). The supernatant obtained was employed to estimate thiobarbituric acid-reactive substances (TBARS) (Ohkawa et al., 1979), reduced glutathione (GSH) (Beutler et al., 1963), catalase (CAT) (Aebi, 1984), and acetylcholinesterase (AChE) (Ellman et al., 1961). Total protein concentration was estimated using the biuret kit method. The observations were made in triplicate.

#### Estimation of thiobarbituric acid-reactive substances

Oxidative stress is a consequence of generation of reactive oxygen species. Some of these species are stable (malondialdehyde) while others are transient (hydroxynonenol) (Miyata and Smith, 1996; Subramaniam et al., 1998; Keller et al., 2000; Pedersen et al., 2000). In determination of TBARS, evaluation of malondialdehyde is particularly important. It is necessary to measure the amount of TBARS for estimation of the antioxidant effect of treatment on brain tissue.

##### Procedure for TBARS estimation in the brain cortex and hippocampus of mice

The supernatant of the tissue homogenate was used to estimate TBARS. In a test tube, 0.2 ml of the homogenate supernatant was pipetted out. Furthermore, 0.2 ml of 8.1% sodium dodecyl sulfate, 1.5 ml of 30% glacial acetic acid (pH 3.5), and 1.5 ml of thiobarbituric acid were added to the test tube, and a final volume of 4 ml was made using distilled water. For 1 h, the test tubes were kept in an incubator at 95°C. After 1 h, the test tubes were taken out from the incubator and the contents were allowed to cool. One ml of distilled water was added followed by addition of 5 ml of 15:1 v/v mixture of n-butanol-pyridine. For 10 min the tubes were centrifuged at 4,000 *g* (at 4°C). The absorbance of the developed pink color of the supernatant was measured using a double beam UV spectrophotometer (Shimadzu, Japan) at 532 nm. A calibration curve was prepared using different nanomolar concentrations of 1,1,3,3 tetramethoxypropane. The TBARS value was expressed as nM/mg of protein (Ohkawa et al., 1979). The observations were made in triplicate.

#### Estimation of reduced glutathione (GSH)

The GSH content in the tissue was estimated using the method of Beutler et al. (1963).

##### Procedure for GSH estimation in the brain cortex and hippocampus of mice

Trichloroacetic acid 10% w/v was added to the homogenate supernatant equal volumes (300 μl of each). For 10 min, the contents of the tubes were centrifuged at 1,000 *g* (at 4°C). After centrifugation, 0.5 ml of the supernatant obtained was added to 2 ml of 0.3 M disodium hydrogen phosphate. Then, a fresh 0.001 M solution of DTNB [5,5-dithiobis (2- nitrobenzoic acid)] was prepared by dissolving it in 0.25 ml of 1% w/v sodium citrate. It was added to the previous solution, and absorbance was noted using the double beam UV spectrophotometer at 412 nm. A standard curve was plotted using a range of serial dilutions of a reduced form of glutathione (10–100 μM), and the results were expressed in μM of the reduced glutathione/mg of protein. The observations were made in triplicate.

#### Estimation of catalase activity

The catalase activity was estimated using the method of Aebi (1974).

##### Procedure for estimation of CAT activity in the brain cortex and hippocampus of mice

Fifty microliters of the homogenate supernatant was added to a cuvette (3 ml) that already contained 1.95 ml of 50 mM phosphate buffer (pH 7). One milliliter of hydrogen peroxide (30 mM) was added to the contents of the cuvette, and changes in absorbance were recorded at intervals of 15 s for a period of 30 s at 240 nm (0, 15, and 30 s. The catalase activity was calculated using the millimolar extinction coefficient of H_2_O_2_ (0.071 mmol cm^–1^), and the activity was expressed as micromoles of H_2_O_2_ oxidized/min/mg protein using the following equation:


$$\mathrm{CAT}\,\mathrm{activity}=\frac{\delta O.D.}{\varepsilon \,X\,V\,X\,\mathrm{mg}\,\mathrm{of}\,\mathrm{protein}}$$

where “δ O.D.” is the change in absorbance/min; “ε” is the extinction coefficient of hydrogen peroxide (0.071 m mol cm^–1^), and “V” is the volume of sample in ml. The observations were made in triplicate.

#### Estimation of AChE activity

The cortical and hippocampal AChE activities were measured with the method of Ellman et al. (1961) with a slight modification (Khurana et al., 2011). This was estimated utilizing the formation of yellow color following the reaction between thiocholine and dithiobisnitrobenzoate ions. The rate at which thiocholine was formed from acetylcholine iodide in the presence of the brain cholinesterase enzyme was measured using the double beam UV spectrophotometer.

##### Procedure for estimation of AChE activity in the brain cortex and hippocampus of mice

One hundred μL of the homogenate supernatant was taken in a test tube. It was diluted using a fresh solution of DTNB (100 mg DTNB in 1,000 ml of Sorenson phosphate buffer; pH 8). The final volume was made 5 ml, which was further split into two parts of 2.5 ml each. Into one of the test tubes containing 2.5 ml of the previous solution, two drops of a donepezil hydrochloride solution were added. One ml of a substrate solution (acetyl choline chloride) was added in both the test tubes. The test tubes with donepezil hydrochloride were used as individual blanks for each set of the samples. The change in the absorption/min of the test sample was recorded using the double beam UV spectrophotometer at 420 nm (at 0, 1, and 2 min). AChE activity was calculated using the following formula:


$$\text{R}\;\text{=}\;\frac{\text{δ}\;\text{O}.D.\;\text{X}\;\text{vol}.\;\text{of}\;\text{assay}}{\text{ε}\;\text{X mg of protein}}$$

where “R” is the rate of enzyme activity in the “n” mole of acetylcholine chloride hydrolyzed/min/mg protein, “δ O.D.” is change in absorbance/min, and “ε” is extinction coefficient (13,600/M/cm). The observations were made in triplicate.

#### Estimation of total protein in the cortex and hippocampus

Total protein was estimated according to the biuret method with a slight modification. The procedure was followed as given in the inserted instructions in the total protein estimation kit by Erba Mannheim (2017).

The following formula is used to calculate the quantity of total proteins:


Total⁢protein⁢(g/dl)=

$$\frac{\mathrm{Absorbance}\,\mathrm{of}\,\mathrm{test}}{\mathrm{Absorbance}\,\mathrm{of}\,\mathrm{standard}}\times \mathrm{Concentration}\,\mathrm{of}\,\mathrm{standard}\,\left(\text{g}/\text{dl}\right)$$

The above formula is used to estimate the total protein in 10% w/v brain homogenate in PBS. The exact quantity of protein was converted to mg/dl weight of brain tissue (i.e., brain cortex and hippocampus). The observations were made in triplicate.

#### Estimation of mouse IL-6 and TNF-α

The supernatant of the mice brain regions was analyzed for estimation of the parameters according to the instructions provided by Raybiotech United States. Mouse IL-6 and TNF-α monoclonal antibodies were pre-coated to all the wells of a microplate. All the reagents were kept at room temperature (25^°^C) before using them. A 100-μl standard and 100-μl supernatant of the cerebral cortex and the hippocampus were pipetted into standard and test wells. It was placed for 2.5 h at room temperature (25^°^C) with gentle shaking. After 2.5 h, the solution was removed and washed 4 times with 300 μl washing buffer to remove unbound antibody enzymes using a pipette and dried properly by inverting a microplate on paper. One hundred microliters of 1 × biotinylated antibody was added to all the wells of the microplate, placed for 1 h at 25^°^C with gentle shaking, and we repeated the above washing step. Streptavidin solution 100 μl was added to all the wells, kept at 25^°^C for 45 min with gentle shaking, and we repeated the above washing step. Furthermore, 100 μl TMB one-step substrate reagent was pipetted to all the wells and placed for 30 min at 250^°^C. A stop solution of 50 μl was put into each well. Immediately, using an ELISA plate reader, the absorbance was measured at a wavelength of 450 nm. The mouse IL-6 and TNF-α concentrations were calculated using a standard plot of mouse IL-6 and TNF-α. This unit was expressed as mouse IL-6 and TNF-α (pg/ml).

### Statistical analysis

The results were reported as mean ± SEM. A two-way analysis of variance (ANOVA) was conducted for statistical analysis of behavioral parameters. A one-way analysis of variance (ANOVA) was also conducted for biochemical estimation. The statistical significance of the results was considered at *P* < 0.05, *P* < 0.01, and *P* < 0.001 values by Tukey’s test. All the statistical analyses were carried out using Sigma Stat 4.0 v.

## Results

### PASS-predicted analysis of vanillin (*in silico*)

The Pa value for various AD related targets as obtained from PASS software have been illustrated in Table 1.

**TABLE 1:** Prediction of potential anti-dementia and antioxidant activities (Pa) of compounds with the PASS software.

Compound	Canonical SMILES (obtained from PubChem)	Predicted pharmacological activity (P_a_ value)					
		
		Anti-amyloidogenic	Anti-Aβ aggregatory	Anti-dementia	Free radical scavenger	Catalase stimulant	Glutathione hydrogenase (ascorbate) inhibitor
Vanillin	COC1 = C[C = CC(= C1)C = O]O	0.114	0.132	0.445	0.546	0.178	0.366

Only probable activity (P_a_) greater than probable inactivity (P_i_) has been considered.

#### *In vitro* acetylcholinesterase inhibitory assay of vanillin

**FIGURE 1:**
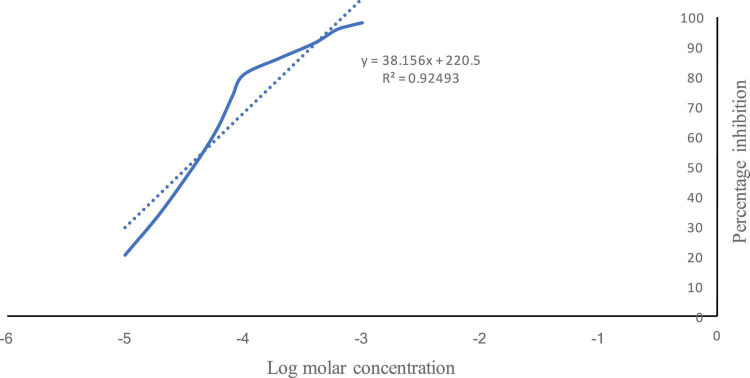
Percentage inhibition of acetylcholinesterase by vanillin. Solid line shows the % inhibition of AChE by vanillin v/s log molar concentration. Dotted line shows the regressed line.

The log molar concentration (x) corresponding to 50% inhibition was −4.47, and the IC_50_ value was calculated to be 0.033 mM (Figure 1).

### *In vivo* study (exteroceptive memory model)

#### Effect of vanillin on locomotor activity

**FIGURE 2:**
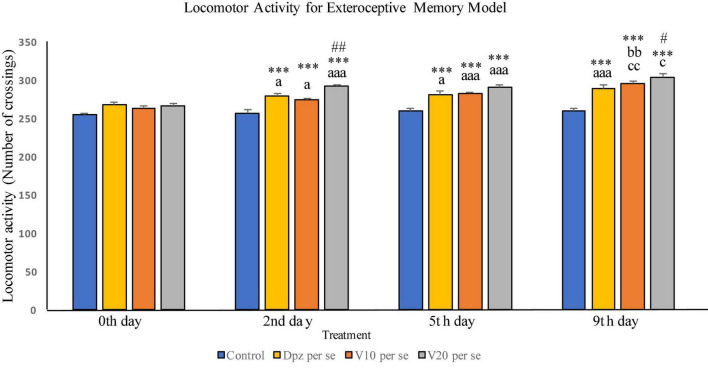
Effect of vanillin and other treatments on locomotor activity. Data are expressed as mean ± SEM. “***” denotes *P* < 0.001 with respect to the vehicle control group within the same day. “##” denotes *P* < 0.01 with respect to the standard treatment group within the same day, and “#” denotes *P* < 0.05 with respect to the standard treatment group within the same day. “aaa” denotes *P* < 0.001 with respect to the same group during the 0th day, and “a” denotes *P* < 0.05 with respect to the same group during 0th day. “bb” denotes *P* < 0.01 with respect to the same group during the 2nd day. “cc” denotes *P* < 0.01 with respect to the same group during the 5th day, and “c” denotes *P* < 0.05 with respect to the same group during the 5th day.

Effect of vanillin and other treatments on locomotor activity in exteroceptive memory model has been illustrated in Figure 2.

##### Locomotor activity on the 0th day

The difference between the locomotor activities of all the treatment groups within the 0th day was not found to be statistically significant.

##### Locomotor activity on the 2nd day

The Dpz *per se* group receiving only donepezil hydrochloride showed a significant (*P* < 0.05) increase in locomotor activity with respect to the same group on the 0th day as well as a significant (*P* < 0.001) increase in locomotor activity with respect to the control group receiving only normal saline within the 2nd day. The V_10_ *per se* group receiving only vanillin (10 mg/kg) exhibited a significant (*P* < 0.05) increase in locomotor activity with respect to the same group on the 0th day as well as a significant (*P* < 0.001) increase in locomotor activity with respect to the control group within the 2nd day. The locomotor activity for the V_20_ *per se* group receiving only vanillin (20 mg/kg) was significantly (*P* < 0.001) increased with respect to the same group on the 0th day, significantly (*P* < 0.001) increased with respect to the control group within the 2nd day, and significantly (*P* < 0.01) increased with respect to the Dpz *per se* group within the 2nd day. Vanillin treatment dose dependently increased the locomotor activity.

##### Locomotor activity (5th day)

The Dpz *per se* group has shown a significant (*P* < 0.05) increase in locomotor activity with respect to the same group on the 0th day. Also, a significant (*P* < 0.001) rise in the locomotor activity was observed in Dpz *per se* treated animals, when compared to the vehicle control group within the 5th day. The V_10_ *per se* group exhibited a significant (*P* < 0.001) increase in locomotor activity with respect to the same group on the 0th day as well as a significant (*P* < 0.001) increase in locomotor activity with respect to the control group within the 5th day. The locomotor activity for the V_20_ *per se* group was significantly (*P* < 0.001) higher with respect to the same group on the 0th day. A significant (*P* < 0.001) increase in the locomotor activity of the V_20_*per se* group was observed with respect to the vehicle control group within the 5th day. Treatment with the vanillin dose dependently increased the locomotor activity.

##### Locomotor activity (9th day)

The locomotor activity for Dpz *per se* group was significantly (*P* < 0.001) higher with respect to the same group on the 0th day as well as significantly (*P* < 0.001) increased than that for the vehicle control group within the 9th day. The V_10_ *per se* group showed a significant (*P* < 0.01) increase in locomotor activity with respect to the same group on the 2nd and 5th days. The vanillin treatment increased the locomotor activity in a dose-dependent manner. Both the vanillin-treated groups, V_10_ *per se* and V_20_*per se*, have shown a significant (*P* < 0.001) increase in the locomotor activity with respect to the control group within the 9th day. The V_20_ *per se* group showed a significantly (*P* < 0.05) higher locomotor activity with respect to the same group on the 5th day. The locomotor activity of the V_20_ *per se* group was significantly (*P* < 0.05) higher than that of the Dpz *per se* group within the 9th day.

#### Effect of vanillin on step-down latency

**FIGURE 3:**
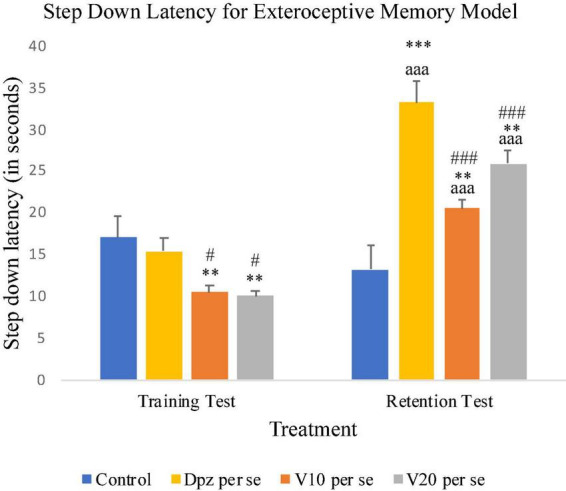
Effect of vanillin and other treatments on step-down latency. Data are expressed as mean ± SEM. “***” denotes *P* < 0.001 with respect to the control group within the same day, and “**” denotes *P* < 0.01 with respect to the control group within the same day. “###” denotes *P* < 0.001 with respect to the standard treatment group within the same day, and “#” denotes *P* < 0.05 with respect to the standard treatment group within the same day. “aaa” denotes *P* < 0.001 with respect to the SDL of the same group on the 10th day of the treatment.

On 10th day, i.e., the training test, the V_10_ *per se* and V_20_ *per se* groups showed significantly (*P* < 0.01) lesser SDL than the control group and significantly (*P* < 0.05) lesser SDL than the Dpz *per se* group. During the retention test, the SDL for the V_10_ *per se* and V_20_ *per se* groups were significantly (*P* < 0.01) increased with respect to the control group. The SDL for the V_10_ *per se* and V_20_ *per se* groups was significantly increased (*P* < 0.001) with respect to the SDL of same groups on the 10th day. The Dpz *per se* group exhibited significantly (*P* < 0.001) higher SDL than the V_10_ *per se* and V_20_ *per se* groups. The vanillin treatment increased the SDL in a dose-dependent manner with respect to the control group (Figure 3).

#### Effect of vanillin on transfer latency

**FIGURE 4:**
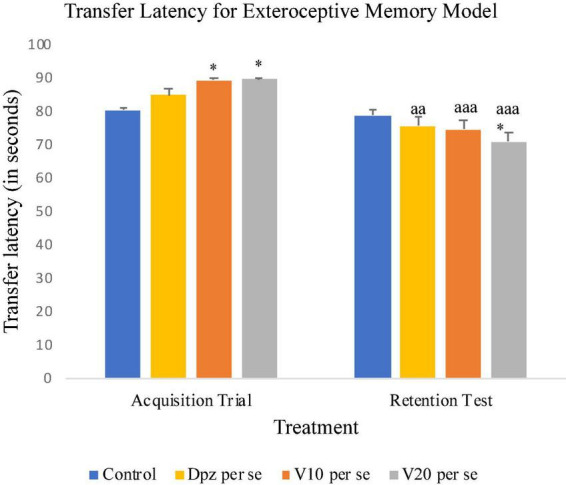
Effect of vanillin and other treatments on transfer latency. Data are expressed as mean ± SEM. “*” denotes *P* < 0.05 with respect to the control group within the same day. “aaa” denotes *P* < 0.001 with respect to the TL of the same group on the 10th day of the treatment, and “aa” denotes *P* < 0.01 with respect to the TL of the same group on the 10th day of the treatment.

On the 10th day, the Dpz *per se*, V_10_ *per se*, and V_20_ *per se* groups showed higher TL than the control group. However, the TL for vanillin-treated groups V_10_ *per se* (receiving vanillin 10 mg/kg) and V_20_ *per se* (receiving vanillin 20 mg/kg) showed a significantly (*P* < 0.05) higher TL than that for the control group. On the 11th day, the V_20_ *per se* group showed a significantly (*P* < 0.05) lesser TL than the control group. The TL for the V_10_ *per se* and V_20_ *per se* groups was significantly lesser (*P* < 0.001) with respect to the same groups on the 10th day. The Dpz *per se* group receiving only donepezil hydrochloride exhibited a significant (*P* < 0.01) decrease in TL with respect to the same group on the 10th day (Figure 4).

#### Effect of vanillin on escape latency

##### Acquisition trials (Figure 5)

###### Training trial

The Dpz *per se* group receiving only donepezil hydrochloride showed a significantly (*P* < 0.01) lesser EL than the control group receiving only normal saline. Vanillin-treated groups V_10_ *per se* (receiving vanillin 10 mg/kg) and V_20_ *per se* (receiving vanillin 20 mg/kg) exhibited a significantly (*P* < 0.001) lower EL than the control group. The EL of the V_20_ *per se* group was significantly (*P* < 0.001) lesser than that of the Dpz *per se* group.

**FIGURE 5:**
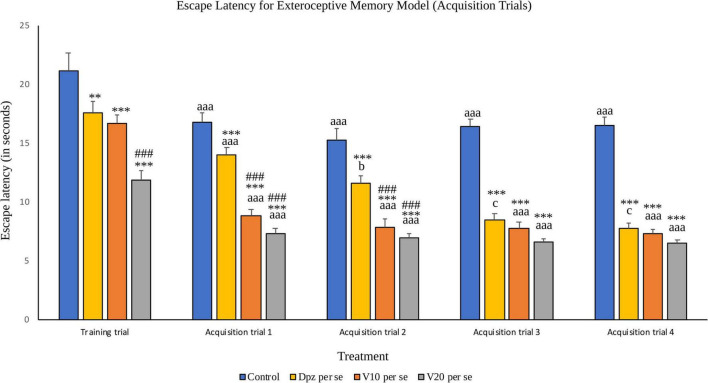
Effect of vanillin and other treatments on escape latency (acquisition trials). Data are expressed as mean ± S.E.M. “***” denotes *P* < 0.001 with respect to the control group within the same day, and “**” denotes *P* < 0.01 with respect to the control group within the same day. “###” denotes *P* < 0.001 with respect to the standard treatment group within the same day. “aaa” denotes (*P* < 0.001) with respect to the acquisition time of the same group during the training trial. “b” denotes *P* < 0.05 with respect to the retention time of the same group during acquisition trial 1, and “c” denotes *P* < 0.05 with respect to the retention time of the same group during acquisition trial 2.

###### Acquisition trial 1

The EL for all the treatment groups within acquisition trial 1 was reduced significantly (*P* < 0.001) with respect to their counterparts during the training trial. The Dpz *per se* group, V_10_ *per se* group, and V_20_*per se* group showed significantly (*P* < 0.001) reduced EL than the control group. The vanillin treatment dose dependently decreased the EL. The EL for both the vanillin-treated groups, V_10_ *per se* and V_20_ *per se*, was significantly (*P* < 0.001) lesser than that for the Dpz *per se* group.

###### Acquisition trial 2

The control group showed a significantly (*P* < 0.001) lower EL than the same group during the training trial. The EL for the Dpz *per se* group was significantly (*P* < 0.05) lesser than the EL for the same group during acquisition trial 1. The EL for the vanillin treated groups, V_10_ *per se* and V_20_ *per se*, was significantly (*P* < 0.001) lesser than that for their counterparts during the training trial. The Dpz *per se* group, V_10_ *per se* group, and V_20_ *per se* group showed significantly (*P* < 0.001) lesser EL than the control group. The treatment with vanillin dose dependently decreased the EL. The EL for both the vanillin treated groups, V_10_ *per se* group and V_20_ *per se* group, was significantly (*P* < 0.001) decreased with respect to the Dpz *per se* group.

###### Acquisition trial 3

The control group showed a significantly (*P* < 0.001) lower EL than the same group during the training trial. The EL for the Dpz *per se* group was significantly (*P* < 0.05) lesser than that for the same group during acquisition trial 2. The El for the groups receiving vanillin treatment, V_10_ *per se* and V_20_ *per se*, was significantly (*P* < 0.001) lesser than that for the same groups during the training trial. The Dpz *per se* group, V_10_ *per se* group, and V_20_ *per se* group exhibited significantly (*P* < 0.001) lower EL than the control group. The vanillin treatment decreased the EL in a dose-dependent manner.

###### Acquisition trial 4

The control group showed a significantly (*P* < 0.001) lower EL than the same group during the training trial. The EL for the Dpz *per se* group was significantly (*P* < 0.05) lesser than that for the same group during acquisition trial 2. The El for the groups receiving vanillin treatment, V_10_ *per se* and V_20_ *per se*, was significantly (*P* < 0.001) lesser than that for the same groups during the training trial. The Dpz *per se* group, V_10_ *per se* group, and V_20_*per se* group exhibited significantly (*P* < 0.001) lower EL than the control group. The treatment with vanillin decreased the EL dose dependently.

###### Probe trial

**FIGURE 6:**
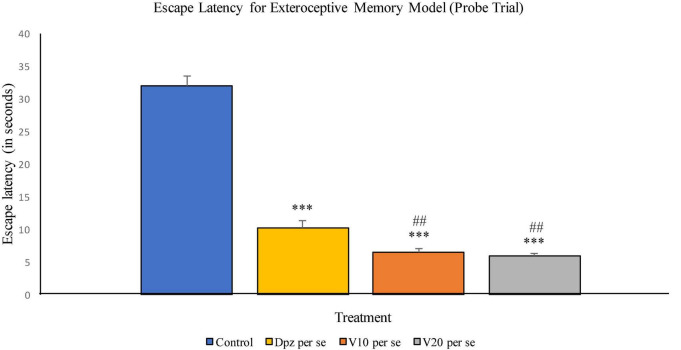
Effect of vanillin and other treatments on escape latency (probe trial). Data are expressed as mean ± SEM. “***” denotes *P* < 0.001 with respect to the control group within the probe trial. “###” denotes *P* < 0.001 with respect to the standard treatment group within the probe trial.

The Dpz *per se* group receiving only donepezil hydrochloride showed a significant (*P* < 0.001) decrease in EL with respect to the control group receiving only normal saline. The vanillin treatment dose dependently decreased the EL in the probe trial. Both groups receiving the vanillin treatment, V_10_ *per se* (10 mg/kg) and V_20_*per se* (20 mg/kg), exhibited a significantly (*P* < 0.001) lesser EL than the control group. The reduction in EL for the V_10_ *per se* and V_20_ *per se* groups was significantly (*P* < 0.01) lesser than that for the Dpz *per se* group (Figure 6).

**FIGURE 7:**
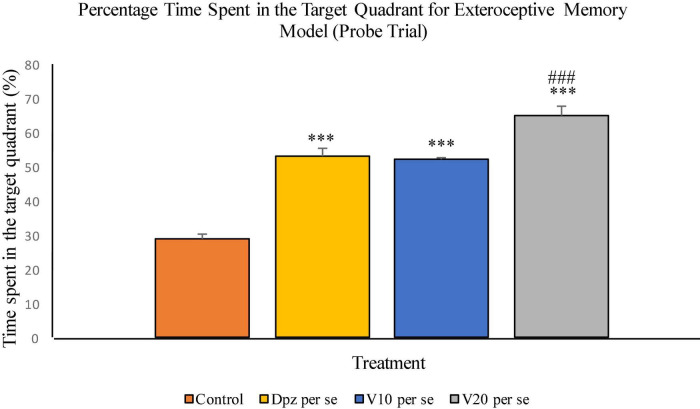
Effect of vanillin and other treatments on percentage time spent in the target quadrant (probe trial). Data are expressed as mean ± SEM. “***” denotes *P* < 0.001 with respect to the control group within the probe trial. “###” denotes *P* < 0.001 with respect to the standard treatment group within the probe trial.

The Dpz *per se* group receiving only donepezil hydrochloride showed a significant (*P* < 0.001) increase in the percentage time spent in the target quadrant with respect to the control group receiving only normal saline. The vanillin treatment dose dependently increased the percentage time spent in the target quadrant in the probe trial. Both the groups receiving vanillin treatment, V_10_ *per se* (10 mg/kg) and V_20_*per se* (20 mg/kg), exhibited a significantly (*P* < 0.001) greater percentage time spent in the target quadrant with respect to the control group. The increase in percentage time spent in the target quadrant for the V_20_ *per se* group was significantly (*P* < 0.01) greater than that for the Dpz *per se* group (Figure 7).

#### Effect of vanillin on TBARS

##### TBARS assay (Figure 8)

###### In the cortex

The vanillin treatment dose dependently decreased the levels of TBARS in the cortex. The V_10_ *per se* group receiving only vanillin (10 mg/kg) showed a significant (*P* < 0.001) reduction in TBARS with respect to the control group receiving only normal saline and the Dpz *per se* group receiving only donepezil hydrochloride. The level of TBARS was significantly (*P* < 0.001) reduced in the V_20_ *per se* group receiving only vanillin (20 mg/kg) with respect to the control group and the Dpz *per se* group. The Dpz *per se* group showed an insignificant increase in the level of TBARS with respect to the control group.

**FIGURE 8:**
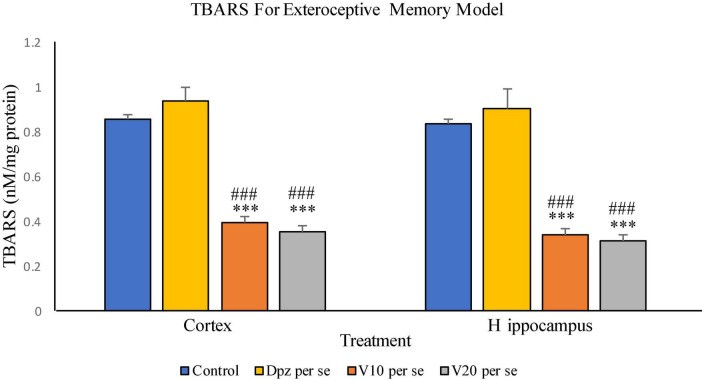
Effect of vanillin and other treatments on TBARS. Data are expressed as mean ± SEM. “***” denotes *P* < 0.001 with respect to the control group. “###” denotes *P* < 0.001 with respect to the standard treatment group.

###### In the hippocampus

The V_10_ *per se* group showed a significant (*P* < 0.001) reduction in TBARS with respect to the control group and the Dpz *per se* group. The level of TBARS was significantly (*P* < 0.001) reduced in the V_20_ *per se* group with respect to the control group and the Dpz *per se* group. The Dpz *per se* group showed an insignificant increase in the level of TBARS with respect to the control group. The vanillin treatment reduced the level of TBARS in the hippocampus in a dose-dependent manner.

#### Effect of vanillin on GSH

##### GSH assay (Figure 9)

###### In the cortex

The Dpz *per se* group receiving only donepezil hydrochloride showed an insignificant increase in the level of GSH with respect to the control group receiving only normal saline. The vanillin treatment dose dependently increased the levels of GSH. The V_10_ *per se* group receiving only vanillin (10 mg/kg) showed a significant (*P* < 0.001) increase in the level of GSH with respect to the control group as well as a significant (*P* < 0.05) increase in the level of GSH with respect to the Dpz *per se* group. The level of GSH was significantly (*P* < 0.001) increased in the V_20_ *per se* group with respect to the control group and the Dpz *per se* group.

**FIGURE 9:**
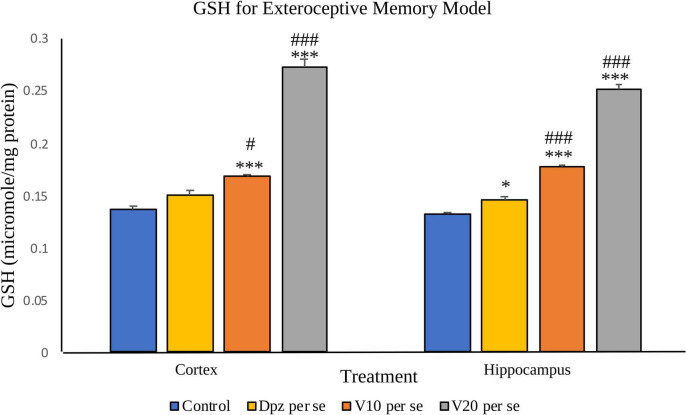
Effect of vanillin and other treatments on GSH. Data are expressed as mean ± SEM. “***” denotes *P* < 0.001 with respect to the control group, and “*” denotes *P* < 0.05 with respect to the control group. “###” denotes *P* < 0.001 with respect to the standard treatment group, and “#” denotes *P* < 0.05 with respect to the standard treatment group.

###### In the hippocampus

The Dpz *per se* group showed a significant (*P* < 0.05) increase in the level of GSH with respect to the control group. The treatment with vanillin increased the levels of GSH in a dose-dependent manner. The V_10_ *per se* group showed a significant (*P* < 0.001) increase in the level of GSH with respect to the control group and the Dpz *per se* group. There was a significant (*P* < 0.001) increase in the level of GSH for the V_20_*per se* group with respect to the control group and the Dpz *per se* group.

#### Effect of vanillin on catalase activity

##### Catalase activity assay (Figure 10)

###### In the cortex

The Dpz *per se* group receiving only donepezil hydrochloride showed an insignificant increase in catalase activity with respect to the control group receiving only normal saline. The V_10_ *per se* group receiving only vanillin (10 mg/kg) showed a significant (*P* < 0.001) increase in the catalase activity with respect to the control group and the Dpz *per se* group. The catalase activity was significantly (*P* < 0.001) increased in the V_20_ *per se* group with respect to the control group and the Dpz *per se* group. The vanillin treatment increased the catalase activity in a dose-dependent manner.

**FIGURE 10:**
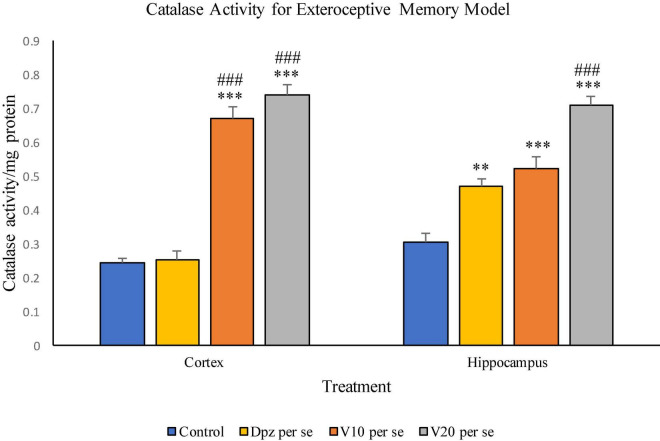
Effect of vanillin and other treatments on catalase activity. Data are expressed as mean ± SEM. “***” denotes *P* < 0.001 with respect to the control group, and “**” denotes *P* < 0.01 with respect to the control group. “###” denotes *P* < 0.001 with respect to the standard treatment group.

###### In the hippocampus

The Dpz *per se* group showed a significant (*P* < 0.01) increase in the catalase activity with respect to the control group. The V_10_ *per se* group exhibited a significant (*P* < 0.001) increase in the catalase activity with respect to the control group. The catalase activity for the V_10_ *per se* group was higher than that for the Dpz *per se* group, but the increase was not significant. The V_20_ *per se* group exhibited a significant (*P* < 0.001) increase in the catalase activity with respect to the control group and the Dpz *per se* group. The treatment with vanillin dose dependently increased the catalase activity.

#### Effect of vanillin on acetylcholinesterase activity

##### AChE activity assay (Figure 11)

###### In the cortex

The Dpz *per se* group receiving only donepezil hydrochloride showed a significant (*P* < 0.05) decrease in AChE activity with respect to the control group receiving only normal saline. The vanillin treatment dependently decreased the activity of AChE. The V_10_ *per se* group receiving only vanillin (10 mg/kg) showed a significant (*P* < 0.01) decrease in AChE activity with respect to the control group. For the V_10_ *per se* group, the AChE activity was lesser than that for the Dpz *per se* group, but the decrease was not significant. A significant (*P* < 0.001) decrease in AChE activity with respect to the control group was observed in the V_20_ *per se* group. The AChE activity for the V_20_ *per se* group was significantly (*P* < 0.05) lesser than that for the Dpz *per se* group.

**FIGURE 11:**
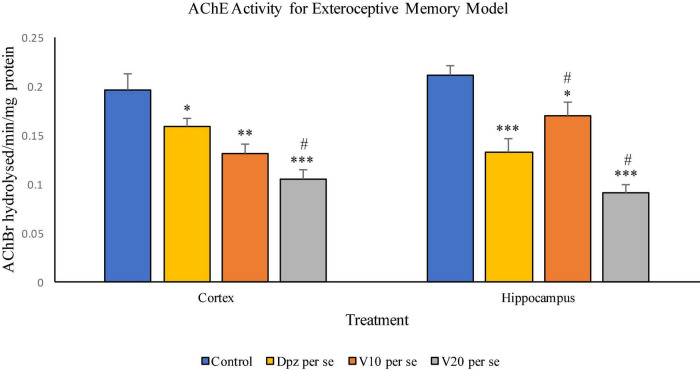
Effect of vanillin and other treatments on AChE activity. Data are expressed as mean ± SEM. “***” denotes *P* < 0.001 with respect to the control group, “**” denotes *P* < 0.01 with respect to the control group, and “* denotes *P* < 0.05 with respect to the control group. “#” denotes *P* < 0.05 with respect to the standard treatment group.

###### In the hippocampus

The Dpz *per se* group showed a significant (*P* < 0.001) decrease in the AChE activity with respect to the control group. The AChE activity for the V_10_ *per se* group was significantly (*P* < 0.05) lesser than that for the control group but significantly (*P* < 0.05) higher than that for the Dpz *per se* group. The V_20_ *per se* group exhibited a significantly (*P* < 0.001) lower AChE activity with respect to the control group. The AChE activity for the V_20_ *per se* group was significantly (*P* < 0.05) lesser than that for the Dpz *per se* group. The vanillin treatment decreased the activity of AChE in a dose-dependent manner.

### *In vivo* study (scopolamine-induced dementia model)

#### Effect of vanillin on locomotor activity

##### Locomotor activity

**FIGURE 12:**
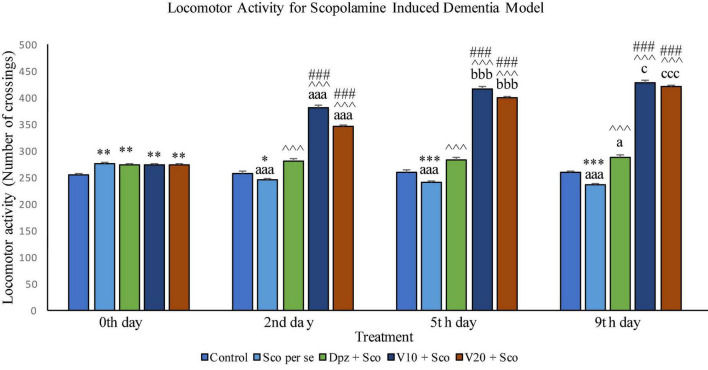
Effect of vanillin and other treatments on locomotor activity. Data are expressed as mean ± SEM. “***” denotes *P* < 0.001 with respect to the control group within the same day, “**” denotes *P* < 0.01 with respect to the control group within the same day, and “*” denotes *P* < 0.05 with respect to the control group within the same day. “###” denotes *P* < 0.001 with respect to the standard treatment group within the same day. “^∧∧∧^” denotes *P* < 0.001 with respect to the negative control group (Sco *per se*) within the same day. “aaa” denotes *P* < 0.001 with respect to the same group during the 0th day, and “a” denotes *P* < 0.05 with respect to the same group during the 0th day. “bbb” denotes *P* < 0.001 with respect to the same group during the 2nd day. “ccc” denotes *P* < 0.001 with respect to the same group during the 5th day, and “c” denotes *P* < 0.05 with respect to the same group during the 5th day.

Effect of vanillin and other treatments on locomotor activity in scopolamine induced dementia model has been illustrated in Figure 12.

###### Locomotor activity on the 0th day

The locomotor activity for all the treatment groups was significantly (*P* < 0.01) greater than that for the control group receiving only normal saline.

###### Locomotor activity on the 2nd day

The locomotor activity for the Sco *per se* group receiving only scopolamine hydrobromide was significantly (*P* < 0.05) lesser than that for the control group. With respect to the same group on the 0th day, the Sco *per se* group had a significant (*P* < 0.001) decrease in the locomotor activity. The Dpz + Sco group receiving donepezil hydrochloride and scopolamine hydrobromide had a significant (*P* < 0.001) increase in the locomotor activity with respect to the Sco *per se* group. The locomotor activity for the V_10_ + Sco group receiving vanillin 10 mg/kg and scopolamine hydrobromide was significantly (*P* < 0.001) increased with respect to the Sco *per se* group and the Dpz + Sco group. The V_20_ + Sco group receiving vanillin 20 mg/kg and scopolamine hydrobromide showed a significant (*P* < 0.001) increase in the locomotor activity with respect to the Sco *per se* group and the Dpz + Sco group. The vanillin treated groups, V_10_ + Sco and V_20_ + Sco, exhibited a significantly (*P* < 0.001) greater locomotor activity than their respective counterparts on the 0th day.

###### Locomotor activity on the 5th day

The Sco *per se* group had a significantly (*P* < 0.001) decreased locomotor activity with respect to the control group. With respect to the same group on the 0th day, the Sco *per se* group had a significantly (*P* < 0.001) decreased locomotor activity. The Dpz + Sco group showed a significantly (*P* < 0.001) increased locomotor activity with respect to the Sco *per se* group. The locomotor activity for the V_10_ + Sco group was significantly (*P* < 0.001) higher with respect to the Sco *per se* group and the Dpz + Sco group. The V_20_ + Sco group exhibited a significant (*P* < 0.001) increase in the locomotor activity with respect to the Sco *per se* group and the Dpz + Sco group. The vanillin-treated groups, V_10_ + Sco and V_20_ + Sco, exhibited a significantly (*P* < 0.001) greater locomotor activity than their respective counterparts on the 2nd day.

###### Locomotor activity on the 9th da*y*

The locomotor activity for the Sco *per se* group was significantly (*P* < 0.001) lesser than that for the control group within the same day and the same group on the 0th day. The Dpz + Sco group had a significant (*P* < 0.001) increase in the locomotor activity with respect to the control group. Also, the Dpz + Sco group showed a significant (*P* < 0.05) increase in the locomotor activity with respect to the same group on the 0th day. The vanillin-treated groups, V_10_ + Sco and V_20_ + Sco, exhibited a significantly (*P* < 0.001) higher locomotor activity than the Sco *per se* group and the Dpz + Sco group. The V_10_ + Sco group showed a significant (*P* < 0.05) increase in the locomotor activity with respect to the same group on the 5th day. The locomotor activity for the V_20_ + Sco group was significantly (*P* < 0.001) increased with respect to the same group on the 5th day.

#### Effect of vanillin on step-down latency

On the 10th day, there was no significant difference in SDL between all the treatment groups except for the V_20_ + Sco group receiving vanillin (20 mg/kg) along with scopolamine hydrobromide, which showed a significantly (*P* < 0.05) lesser SDL than the control group receiving only normal saline.

**FIGURE 13:**
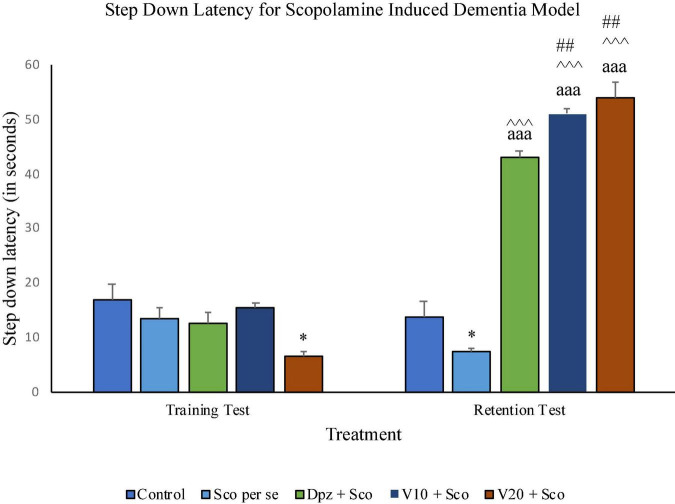
Effect of vanillin and other treatments on step-down latency. Data are expressed as mean ± SEM. “*” denotes *P* < 0.05 with respect to the control group within the same day. “##” denotes *P* < 0.01 with respect to the standard treatment group within the same day. “^∧∧∧^” denotes *P* < 0.001 with respect to the negative control group (Sco *per se*) within the same day. “aaa” denotes *P* < 0.001 with respect to the SDL of the same group on the 10th day of the treatment.

During the training test on the 11th day, the Sco *per se* group had a significant (*P* < 0.05) decrease in the SDL with respect to the control group. The Dpz + Sco, V_10_ + Sco, and V_20_ + Sco groups exhibited a significant (*P* < 0.001) increase in SDL with respect to the Sco *per se* group. The vanillin treatment dose dependently increased the SDL. The V_10_ + Sco group and the V_20_ + Sco group showed a significant (*P* < 0.01) increase in SDL with respect to the Dpz + Sco group. The SDL for the Dpz + Sco group, V_10_ + Sco group, and V_20_ + Sco group was significantly (*P* < 0.001) increased with respect to their respective counterparts on the 10th day (Figure 13).

#### Effect of vanillin on transfer latency

**FIGURE 14:**
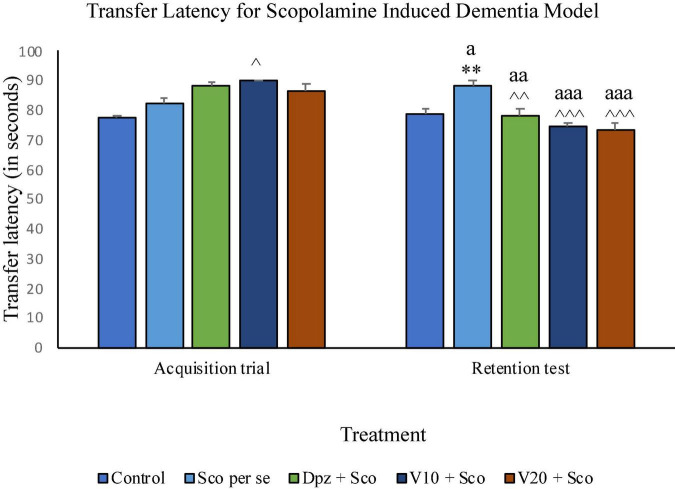
Effect of vanillin and other treatments on transfer latency. Data are expressed as mean ± SEM. “**” denotes *P* < 0.01 with respect to the control group within the same day. “^∧∧∧^” denotes *P* < 0.001 with respect to the negative control group (Sco *per se*) within the same day. “^∧∧^” denotes *P* < 0.01 with respect to the negative control group (Sco *per se*) within the same day. “^∧^” denotes *P* < 0.05 with respect to the negative control group (Sco *per se*) within the same day. “aaa” denotes *P* < 0.001 with respect to the TL of the same group on the 10th day of the treatment, and “aa” denotes *P* < 0.01 with respect to the TL of the same group on the 0th day of the treatment.

On the 10th day, there was no significant difference between the TL of the Sco *per se* group receiving only scopolamine hydrobromide and the control group receiving only normal saline. The V_10_ + Sco group receiving vanillin (10 mg/kg) and scopolamine hydrobromide showed a significant (*P* < 0.05) increase in TL with respect to the Sco *per se* group. On the 11th day, the TL for the Sco *per se* group was significantly (*P* < 0.01) higher than that for the control group. The Dpz + Sco group receiving donepezil hydrochloride and scopolamine had a significantly (*P* < 0.01) decreased TL with respect to the Sco *per se* group. The vanillin-treated groups, V_10_ + Sco (vanillin 10 mg/kg + scopolamine hydrobromide) and V_20_ + Sco (vanillin 20 mg/kg + scopolamine hydrobromide) exhibited a TL significantly (*P* < 0.001) lesser than that of the Sco *per se* group. The Dpz + Sco group had a significant (*P* < 0.01) reduction in TL with respect to the same group on the 10th day. The V_10_ + Sco group and the V_20_ + Sco group showed a significantly (*P* < 0.001) decreased TL with respect to their respective counterparts on the 10th day (Figure 14).

#### Effect of vanillin on escape latency

##### Acquisition trials (Figure 15)

###### Training trial

The Sco *per se* group receiving only scopolamine hydrobromide had a significantly (*P* < 0.001) higher EL with respect to the control group. The Dpz + Sco group receiving donepezil hydrochloride and scopolamine hydrobromide exhibited a significantly (*P* < 0.001) lower EL with respect to the Sco *per se* group. The vanillin-treated groups, V_10_ + Sco (vanillin 10 mg/kg + scopolamine hydrobromide) and V_20_ + Sco (vanillin 20 mg/kg + scopolamine hydrobromide), showed a significantly (*P* < 0.001 lower EL with respect to the Sco *per se* group. The EL for the V_10_ + Sco group was significantly (*P* < 0.01) higher than that for the Dpz + Sco group.

**FIGURE 15:**
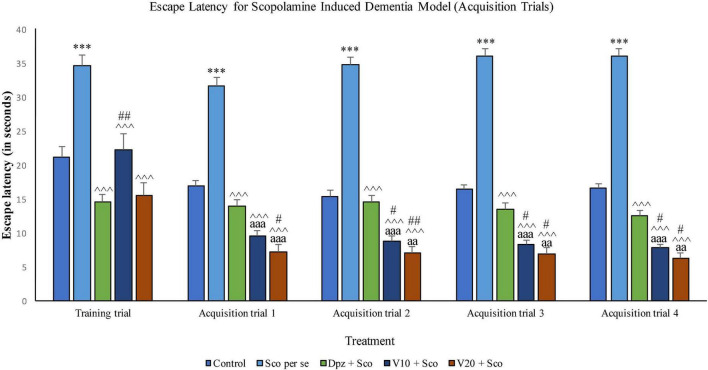
Effect of vanillin and other treatments on escape latency (acquisition trials). Data are expressed as mean ± SEM. “***” denotes *P* < 0.001 with respect to the control group within the same day. “##” denotes *P* < 0.01 with respect to the standard treatment group within the same day, and “#” denotes *P* < 0.05 with respect to the standard treatment group within the same day. “^∧∧∧^” denotes *P* < 0.001 with respect to the negative control group (Sco *per se*). “aaa” denotes *P* < 0.001 with respect to the acquisition time of the same group during the training trial, and “aa” denotes *P* < 0.01 with respect to the acquisition time of the same group during the training trial.

###### Acquisition trial 1

The Sco *per se* group exhibited a significantly (*P* < 0.001) higher EL with respect to the control group. The Dpz + Sco group, V_10_ + Sco group, and V_20_ + Sco group had a significantly (*P* < 0.001) decreased EL with respect to the Sco *per se* group. The vanillin treatment dose dependently decreased the EL. The V_10_ + Sco group and the V_20_ + Sco group showed a significantly (*P* < 0.001) lower EL than their respective counterparts during the training trial. The V_20_ + Sco group had a significantly (*P* < 0.05) lower TL with respect to the Dpz + Sco group.

###### Acquisition trial 2

The EL for the Sco *per se* group was significantly (*P* < 0.001) greater than that for the control group. The Dpz + Sco group, V_10_ + Sco group, and V_20_ + Sco group had a significantly (*P* < 0.001) reduced EL with respect to the Sco *per se* group. The vanillin treatment decreased the EL in a dose-dependent manner. The EL for the V_10_ + Sco group was significantly (*P* < 0.001) lesser than that for the same group during the training trial. Also, the EL for the V_10_ + Sco group was significantly (*P* < 0.05) lesser than that for the Dpz + Sco group. A minimum EL was observed for the V_20_ + Sco group, which was significantly (*P* < 0.01) lesser than that for the Dpz + Sco group. Also, the EL was significantly (*P* < 0.01) decreased with respect to the same group during the training trial.

###### Acquisition trial 3

The EL for the Sco *per se* group was significantly (*P* < 0.001) higher than that for the control group. The Dpz + Sco group, V_10_ + Sco group, and V_20_ + Sco group had a significantly (*P* < 0.001) lesser EL with respect to the Sco *per se* group. The vanillin treatment decreased the EL dose dependently. The EL for the V_10_ + Sco group was significantly (*P* < 0.001) lesser than that for the same group during the training trial. Also, the EL for the V_10_ + Sco group was significantly (*P* < 0.05) lesser than that for the Dpz + Sco group. A minimum EL was observed for the V_20_ + Sco group, which was significantly (*P* < 0.05) lesser than that for the Dpz + Sco group. Also, the EL was significantly (*P* < 0.01) decreased with respect to the same group during the training trial.

###### Acquisition trial 4

The EL for the Sco *per se* group was significantly (*P* < 0.001) greater than that for the control group. The Dpz + Sco group, V_10_ + Sco group, and V_20_ + Sco group had a significantly (*P* < 0.001) lesser EL with respect to the Sco *per se* group. The vanillin treatment decreased the EL dose dependently. The EL for the V_10_ + Sco group was significantly (*P* < 0.001) decreased than that for the same group during the training trial. Also, the EL for the V_10_ + Sco group was significantly (*P* < 0.05) lesser than for the Dpz + Sco group. A minimum EL was observed for the V_20_ + Sco group, which was significantly (*P* < 0.05) lesser than that for the Dpz + Sco group. Also, the EL was significantly (*P* < 0.01) lesser with respect to the same group during the training trial.

###### Probe trial

**FIGURE 16:**
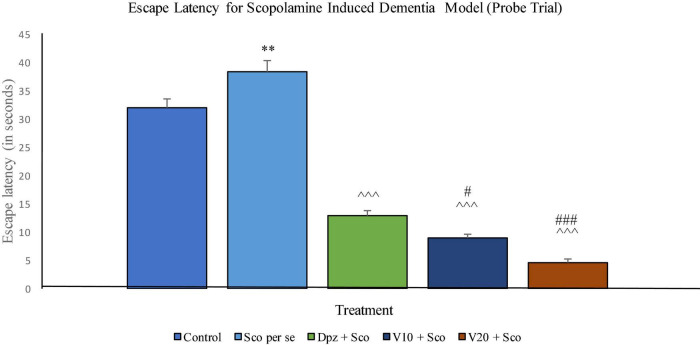
Effect of vanillin and other treatments on escape latency (probe trial). Data are expressed as mean ± SEM. “**” denotes *P* < 0.01 with respect to the control group within the probe trial. “###” denotes *P* < 0.001 with respect to the standard treatment group within the probe trial, “#” denotes *P* < 0.05 with respect to the standard treatment group within the probe trial. “^∧∧∧^” denotes *P* < 0.001 with respect to the negative control (Sco *per se*) group within the probe trial.

The EL was significantly (*P* < 0.001) increased for the Sco *per se* group with respect to the control group. The Dpz + Sco group, V_10_ + Sco group, and V_20_ + Sco group showed a significantly (*P* < 0.001) lesser EL with respect to the Sco *per se* group. The vanillin treatment dose dependently decreased the EL. The V_10_ + Sco group had an EL significantly (*P* < 0.05) lesser than that of the Dpz + Sco group. A minimum EL was observed for the V_20_ + Sco group, which was significantly (*P* < 0.001) lesser than that for the Dpz + Sco group (Figure 16).

**FIGURE 17:**
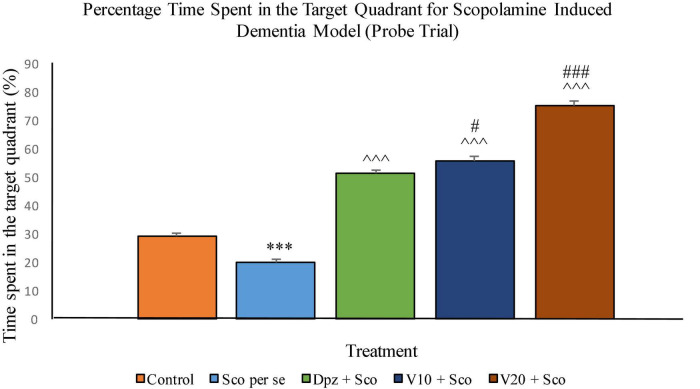
Effect of vanillin and other treatments on percentage time spent in the target quadrant (probe trial). Data are expressed as mean ± SEM. “***” denotes *P* < 0.001 with respect to the control group within the probe trial. “###” denotes *P* < 0.001 with respect to the standard treatment group within the probe trial, and “#” denotes *P* < 0.05 with respect to the standard treatment group within the probe trial. “^∧∧∧^” denotes *P* < 0.001 with respect to the negative control (Sco *per se*) group within the probe trial.

The Sco *per se* group had a significantly (*P* < 0.001) lesser percentage time spent in the target quadrant. The Dpz + Sco group, V_10_ + Sco group, and V_20_ + Sco group showed a significantly (*P* < 0.001) greater percentage time spent in the target quadrant with respect to the Sco *per se* group. The vanillin treatment increased the percentage time spent in the target quadrant in a dose-dependent manner. The V_10_ + Sco group showed a significantly (*P* < 0.05) higher percentage time spent in the target quadrant with respect to the Dpz + Sco group. The maximum percentage time spent in the target quadrant was observed for the V_20_ + Sco group, which was significantly (*P* < 0.001) greater than that for the Dpz + Sco group (Figure 17).

#### Effect of vanillin on TBARS

##### TBARS assay (Figure 18)

###### In the cortex

The Sco *per se* group receiving only scopolamine hydrobromide had significantly (*P* < 0.001) increased TBARS with respect to the control group receiving only normal saline. The Dpz + Sco group receiving donepezil hydrochloride along with scopolamine hydrobromide showed a significant (*P* < 0.05) decrease in TBARS with respect to the Sco *per se* group. The vanillin treatment dose dependently decreased TBARS. The V_10_ + Sco group receiving vanillin 10 mg/kg along with scopolamine hydrobromide exhibited significantly (*P* < 0.001) lower TBARS with respect to the Sco *per se* group as well as the Dpz + Sco group. The level of TBARS was minimum for the V_20_ + Sco group receiving vanillin 20 mg/kg along with scopolamine hydrobromide. The TBARS for the V_20_ + Sco group was significantly (*P* < 0.001) lesser with respect to the Sco *per se* group as well as the Dpz + Sco group.

**FIGURE 18:**
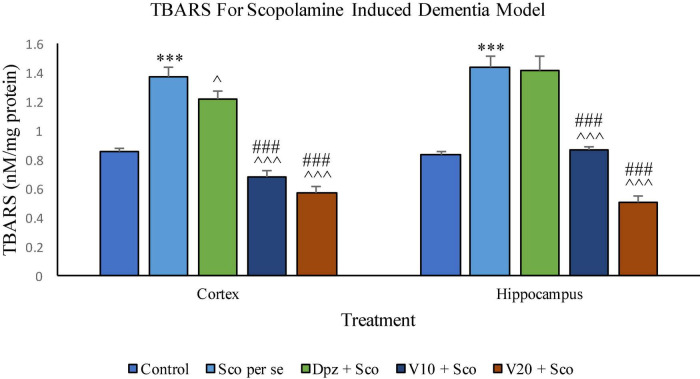
Effect of vanillin and other treatments on TBARS. Data are expressed as mean ± SEM. “***” denotes *P* < 0.001 with respect to the control group. “###” denotes *P* < 0.001 with respect to the standard treatment group. “^∧∧∧^” denotes *P* < 0.001 with respect to the negative control group (Sco *per se*), and “^∧^” denotes *P* < 0.05 with respect to the negative control group.

###### In the hippocampus

The level of TBARS was significantly (*P* < 0.001) higher in the Sco *per se* group with respect to the control group. The Dpz + Sco group showed an insignificant reduction in TBARS with respect to the Sco *per se* group. The vanillin treatment decreased TBARS in a dose-dependent manner. The V_10_ + Sco group showed significantly (*P* < 0.001) decreased TBARS with respect to the Sco *per se* group as well as the Dpz + Sco group. A minimum level of TBARS was observed for the V_20_ + Sco group, which was significantly (*P* < 0.001) lesser with respect to the Sco *per se* group as well as the Dpz + Sco group.

#### Effect of vanillin on GSH

##### GSH assay (Figure 19)

###### In the cortex

The Sco *per se* group receiving only scopolamine hydrobromide showed a significantly (*P* < 0.001) decreased GSH with respect to the control group receiving only normal saline. The Dpz + Sco group receiving donepezil hydrochloride along with scopolamine hydrobromide had an insignificant increase in GSH with respect to Sco *per se* group. The treatment with vanillin increased GSH in a dose-dependent manner. GSH was significantly (*P* < 0.001) increased for the V_10_ + Sco group receiving vanillin (10 mg/kg) along with scopolamine hydrobromide with respect to the Sco *per se* group. Also, the V_10_ + Sco group exhibited a significantly (*P* < 0.001) higher GSH than the Dpz + Sco group. A maximum level of GSH was observed for the V_20_ + Sco group receiving vanillin (20 mg/kg) along with scopolamine hydrobromide, which was significantly (*P* < 0.001) greater than that for the Sco *per se* group and Dpz + Sco group.

**FIGURE 19:**
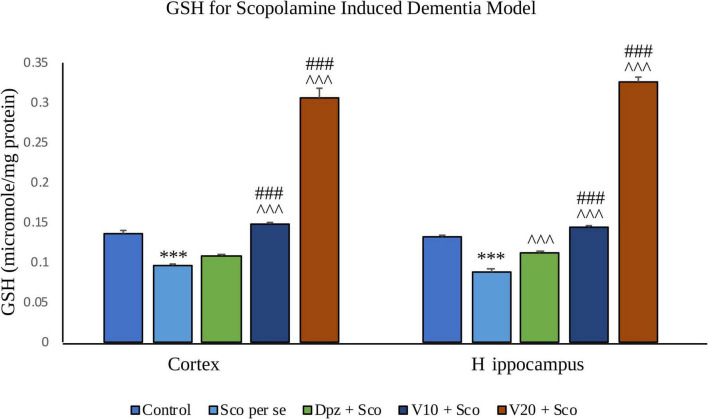
Effect of vanillin and other treatments on GSH. Data are expressed as mean ± SEM. “***” denotes *P* < 0.001 with respect to the control group. “###” denotes *P* < 0.001 with respect to the standard treatment group, and “^∧∧∧^” denotes *P* < 0.001 with respect to the negative control (Sco *per se*) group.

###### In the hippocampus

GSH was significantly (*P* < 0.001) decreased in the Sco *per se* group with respect to the control group. The Dpz + Sco group showed a significant (*P* < 0.001) increase in GSH with respect to the Sco *per se* group. The vanillin treatment dose dependently increased GSH. The vanillin-treated groups, V_10_ + Sco and V_20_ + Sco, had significantly (*P* < 0.001) higher GSH with respect to the Sco *per se* group and the Dpz + Sco group.

#### Effect of vanillin on catalase activity

##### Catalase activity assay (Figure 20)

###### In the cortex

The Sco *per se* group receiving only scopolamine hydrobromide had a significantly (*P* < 0.05) lesser catalase activity with respect to the control group receiving only normal saline. The Dpz + Sco group receiving donepezil hydrochloride along with scopolamine hydrobromide showed a significant (*P* < 0.001) increase in the catalase activity with respect to the Sco *per se* group. The vanillin treatment dose dependently increased the catalase activity. The V_10_ + Sco group receiving vanillin 10 mg/kg and scopolamine hydrobromide exhibited a significantly (*P* < 0.001) increased catalase activity with respect to the Sco *per se* group and the Dpz + Sco group. A maximum catalase activity was observed for the V_20_ + Sco group receiving vanillin 20 mg/kg along with scopolamine hydrobromide, which was significantly (*P* < 0.001) higher than that for the Sco *per se* group and the Dpz + Sco group.

**FIGURE 20:**
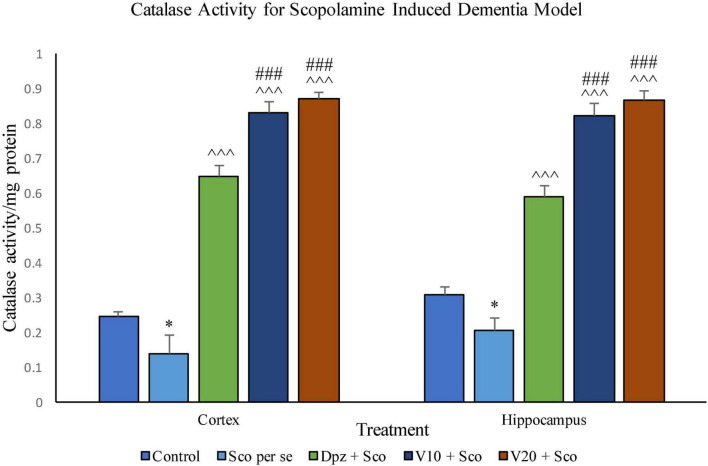
Effect of vanillin and other treatments on catalase activity. Data are expressed as mean ± SEM. “*” denotes *P* < 0.05 with respect to the control group. “###” denotes *P* < 0.001 with respect to the standard treatment group, and “^∧∧∧^” denotes *P* < 0.001 with respect to the negative control (Sco *per se*) group.

###### In the hippocampus

Catalase activity was significantly (*P* < 0.05) decreased in the Sco *per se* group with respect to the control group. The Dpz + Sco group, V_10_ + Sco group, and V_20_ + Sco group exhibited a significantly (*P* < 0.001) higher catalase activity with respect to the Sco *per se* group. The vanillin treatment increased the catalase activity in a dose-dependent manner. The vanillin-treated groups, V_10_ + Sco and V_20_ + Sco, showed a significantly (*P* < 0.001) increased catalase activity with respect to the Dpz + Sco group.

#### Effect of vanillin on acetylcholinesterase activity

##### AChE activity assay (Figure 21)

###### In the cortex

The Sco *per se* group receiving only scopolamine hydrobromide had a significantly (*P* < 0.001) increased AChE activity with respect to the control group receiving only normal saline. The Dpz + Sco group receiving donepezil hydrochloride and scopolamine hydrobromide showed a significant (*P* < 0.001) decrease in AChE activity with respect to the Sco *per se* group. The vanillin treatment dose dependently decreased the AChE activity. The vanillin-treated groups, V_10_ + Sco (vanillin 10 mg/kg + scopolamine hydrobromide) and V_20_ + Sco (vanillin 20 mg/kg + scopolamine hydrobromide), exhibited a significantly (*P* < 0.001) lesser AChE activity with respect to the Sco *per se* group. However, the AChE activity was decreased insignificantly for the V_10_ + Sco group and the V_20_ + Sco group with respect to the Dpz + Sco group.

**FIGURE 21:**
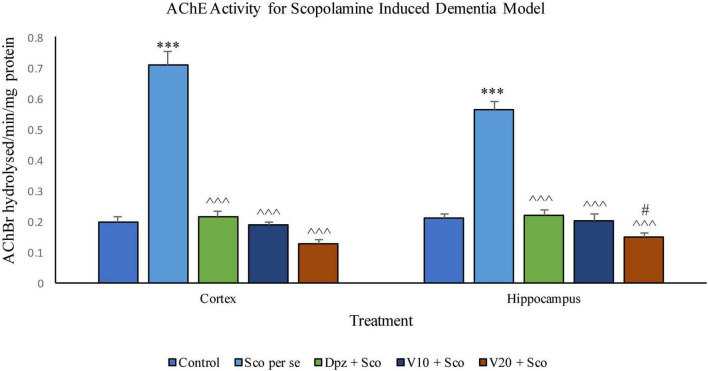
Effect of vanillin and other treatments on AChE activity. Data are expressed as mean ± SEM. “***” denotes *P* < 0.001 with respect to the control group. “#” denotes *P* < 0.05 with respect to the standard treatment group. “^∧∧∧^” denotes *P* < 0.001 with respect to the negative control (Sco *per se*) group.

###### In the hippocampus

AChE activity was significantly (*P* < 0.001) increased in the Sco *per se* group with respect to the control group. The Dpz + Sco group, V_10_ + Sco group, and V_20_ + Sco group showed a significant (*P* < 0.001) decrease in AChE activity with respect to the Sco *per se* group. The vanillin treatment decreased the AChE activity in a dose-dependent manner. A minimum AChE activity was observed for the V_20_ + Sco group, which was significantly (*P* < 0.05) lesser than that for the Dpz + Sco group.

#### Effect of vanillin on IL-6

##### IL-6 concentration assay

###### In the cortex and hippocampus

**FIGURE 22:**
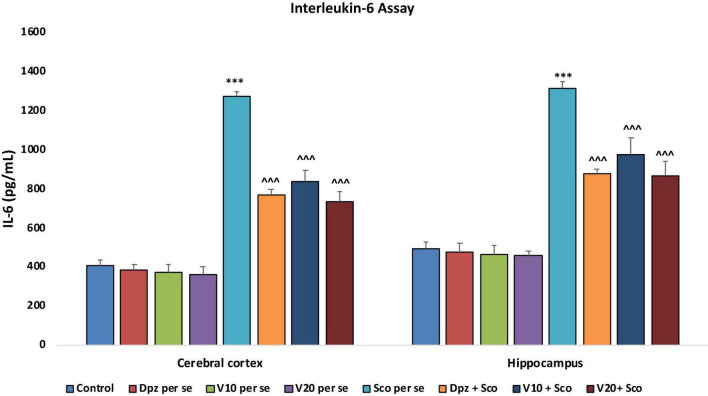
Effect of vanillin and other treatments on IL-6 concentrations. Data are expressed as mean ± SEM. “***” denotes *P* < 0.001 with respect to the control group. “^∧∧∧^” denotes *P* < 0.001 with respect to the negative control (Sco *pe*r se) group.

The Dpz *per se*, V_10_ *per se*, and V_20_*per se* groups showed a slight decrease in the levels of IL-6 as compared to the control group. However, the decrease was not statistically significant. The Sco *per se* group receiving only scopolamine hydrobromide had a significantly (*P* < 0.001) increased IL-6 concentration with respect to the control group receiving only normal saline. The Dpz + Sco group receiving donepezil hydrochloride and scopolamine hydrobromide showed a significant (*P* < 0.001) decrease in IL-6 concentration with respect to the Sco *per se* group. The vanillin treatment dose dependently decreased the IL-6 concentration. The vanillin-treated groups, V_10_ + Sco (vanillin 10 mg/kg + scopolamine hydrobromide) and V_20_ + Sco (vanillin 20 mg/kg + scopolamine hydrobromide), exhibited significantly (*P* < 0.001) lesser IL-6 concentrations with respect to the Sco *per se* group (Figure 22).

#### Effect of vanillin on TNF-α

##### TNF-α concentration assay

###### In the cortex and hippocampus

**FIGURE 23:**
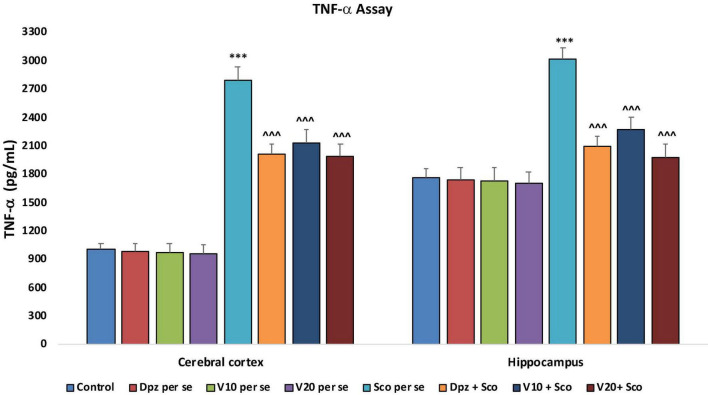
Effect of vanillin and other treatments on TNF-α concentrations. Data are expressed as mean ± SEM “***” denotes *P* < 0.001 with respect to the control group. “^∧∧∧^” denotes *P* < 0.001 with respect to the negative control (Sco *per se*) group.

The Dpz *per se*, V_10_ *per se*, and V_20_ *per se* groups showed a relative decrease in the levels of TNF-α as compared to the control group. However, the decrease was not statistically significant. The Sco *per se* group receiving only scopolamine hydrobromide had a significantly (*P* < 0.001) increased TNF-α concentration with respect to the control group receiving only normal saline. The Dpz + Sco group receiving donepezil hydrochloride and scopolamine hydrobromide showed a significant (*P* < 0.001) decrease in TNF-α concentration with respect to the Sco *per se* group. The vanillin treatment dose dependently decreased the TNF-α concentration. The vanillin-treated groups, V_10_ + Sco (vanillin 10 mg/kg + scopolamine hydrobromide) and V_20_ + Sco (vanillin 20 mg/kg + scopolamine hydrobromide), exhibited significantly (*P* < 0.001) lesser TNF-α concentrations with respect to the Sco *per se* group (Figure 23).

## Discussion

In the modern age, technology plays a major role in all aspects of science. Computers have become an integral part of research projects worldwide. Pharmacology greatly involves the usage of computers and technology. *In silico* studies form the beginning of any drug discovery and development initiative. One such tool for *in silico* studies is PASS Online. Numerous studies have employed PASS Online to predict the probable activity of various compounds, including phytoconstituents, and correlated the findings to the *in vivo* results (Marwaha et al., 2007; Goel et al., 2010; Khurana et al., 2011; Habibyar et al., 2016). In the present study, vanillin was chosen as the test compound because of its PASS-predicted anti-dementia activity, anti-amyloidogenic property, and anti-Aβ aggregatory effect. Also, on the basis of extensive literature review, it was found that vanillin has a good *in vivo* antioxidant property (Dhanalakshmi et al., 2015) and a good anti Aβ aggregatory effect *in vitro* (Song et al., 2016). Oxidative stress has been known to play a pivotal role in the development of AD and related neurodegeneration (Huang et al., 2016). Therefore, in the light of the available data about vanillin, it was selected to be evaluated for its nootropic, anti-dementia, and neuroprotective roles *in vivo*.

In AD, the expression of AChE is increased in the brain and the level of ACh is decreased. This leads to impairment in the memory and cognitive processes (Francis, 2005; Mehta et al., 2012; Singh et al., 2013). Therefore, AChE inhibitory activity is desirable for a compound to be successfully used in the therapy of AD. Therefore, *in vitro* analysis of acetylcholinesterase activity was employed to establish the possible role of vanillin in the treatment of AD. The *in vitro* evaluation of acetylcholinesterase activity of vanillin was made using modified Ellman’s method (Ellman et al., 1961; Atta-ur-Rahman and Choudhary, 2001; Hasnat et al., 2013; Habibyar et al., 2016). Vanillin was found to have an inhibitory effect on acetylcholinesterase with an IC_50_ value of 0.033 mM.

The *in vivo* study was divided into two parts, i.e., exteroceptive memory model and the scopolamine-induced dementia model. The exteroceptive memory model aims at evaluating the nootropic effect of the test compound (Parle et al., 2004; Joshi and Parle, 2006; Vasudevan and Parle, 2008). The pharmacotherapy of disorders involving cognitive impairments such as AD, amnesia, and attention deficit is still a challenge for researchers worldwide. Marketed nootropic drugs, e.g., piracetam, donepezil, aniracetam, have been in use to alleviate memory, behavioral and mood disorders, but their prescription is limited because of associated side effects. The present study was designed to assess the nootropic, i.e., memory-enhancing effect of vanillin with respect to the standard treatment with donepezil hydrochloride. The enhancement in memory was evaluated by behavioral and biochemical analyses. The behavioral studies comprised of assessment of step-down latency in a negative reinforcement paradigm and transfer latency using an elevated plus maze apparatus. The biochemical parameters were assessed for the cortical and hippocampal regions of the brain.

The assessment of locomotor activity was conducted prior to the commencement of the treatment, during the conduct of the treatment, and toward the conclusion of the treatment. The evaluation of locomotor activity was a noteworthy part of the protocol design so as to ascertain the validity of the results obtained for SDL and TL. Any elevation or diminution in the locomotor activity may result in a conflict in the elevated plus maze and negative reinforcement paradigms alike. The donepezil treatment and vanillin treatment both led to increase in locomotor activity as the protocol progressed. The vanillin treatment at the dose of 20 mg/kg showed a significant increase in the locomotor activity with respect to the donepezil *per se* group. The finding was in accordance to the previously reported effect of vanillin on locomotor activity (Gupta and Sharma, 2014; Abdulrahman et al., 2017). Therefore, to evaluate the memory-enhancing potential of vanillin in comparison to donepezil independent of changes in the locomotor activity, the Morris Water Maze paradigm was put to use. Escape from water generally does not depend on differences in activity or body mass; therefore, it can be considered to be appropriate for several experimental models. Also, the odor trail bias is eliminated in the MWM paradigm (Vorhees and Williams, 2006). The findings suggested that vanillin possesses a nootropic effect.

Scopolamine-induced dementia is the commonly used model to assess and evaluate potential anti-AD drug candidates. Scopolamine is a parasympatholytic agent that acts as a competitive muscarinic blocker (Blokland et al., 2006; Kwon et al., 2009). The extent of amelioration or improvement in behavioral parameters with the test molecule based on consistent training and evaluation is utilized as the criterion to report the test compound as to having a potential anti-AD effect. Apart from causing memory and cognitive impairments, scopolamine also leads to oxidative stress, which in turn damages cholinergic neurons. In the present study, the acute and chronic effects of scopolamine, vanillin, and donepezil on the behavioral parameters were evaluated. Also, the effect of vanillin and donepezil on the cortical and hippocampal biochemical parameters (TBARS, GSH, catalase activity, and acetylcholinesterase inhibitory activity) was evaluated. The effect of vanillin on molecular modulators of AD development and progression such as IL-6 and TNF-α was also evaluated. The findings suggested a significant role of vanillin in reversing the deficits in memory and cognition caused by scopolamine.

The findings of the present study clearly indicate the role of vanillin in alleviating oxidative stress, enhancing memory and nootropic activity, and reversing behavioral deficits caused by scopolamine. Vanillin showed a good acetylcholinesterase inhibitory activity *in vitro* and *in vivo*, with the activity being better than that of the donepezil hydrochloride *in vivo*.

Vanillin, a phytoconstituent, has seen light of the day in various research setups. In this study, vanillin has been found to have an *in vitro* acetylcholinesterase inhibitory activity, which was further confirmed by *in vivo* results. Vanillin has also been found to be having a remarkable antioxidant effect. The ease of availability and the established safety of vanillin may contribute to its development as a potential clinical candidate for AD pharmacotherapeutics. However, further research and mechanistic studies are required.

## Conclusion

With the increasing prevalence of AD and other neurodegenerative disorders, the need for more efficacious, safer, and cost-effective pharmacological interventions is greater than ever before. Vanillin is a compound that has been used over the years in multiple ways, and it speaks volumes about its acceptance and safety with respect to consumption by humans. In the present study, the pharmacological role of vanillin in AD has been explored by *in silico*, *in vitro*, and *in vivo* evaluations. It has shown promising results, which were reflected by its capability to control and/or reverse the behavioral deficits caused by the disease-inducing agent, i.e., scopolamine. Not only the behavioral parameters but the ameliorative effect signified by biochemical evaluations of various parameters like AChE, TBARS, GSH, and catalase activity also strengthened the potential anti-AD effect of vanillin. It was also found to significantly decrease the levels of IL-6 and TNF-α in the cortical and hippocampal regions of the brain. This study creates a future pathway for more extensive *in vivo* evaluations of vanillin for its anti-AD effect.

## Data availability statement

The raw data supporting the conclusions of this article will be made available by the authors, without undue reservation.

## Ethics statement

The animal study was reviewed and approved by Institutional Animal Ethics Committee, Lovely Professional University, Phagwara, Punjab, India.

## Author contributions

AA, NK, and NS contributed to the conception and design of the study. AA conducted the study under the supervision of NK and NS. AA performed the statistical analysis and wrote the first draft of the manuscript. NA, AFA, MA, and MW wrote sections of the manuscript and proofread the manuscript. All authors contributed to manuscript revision, read, and approved the submitted version.

## Abbreviations

Ach: acetylcholine
AChE: acetylcholinesterase
AD: Alzheimer’s disease
ANOVA: analysis of variance
GSH: reduced glutathione
SMILES: simplified molecular-input line-entry system
TBARS: thiobarbituric acid-reactive substances
IL-6: Interleukin-6
TNF-α: tumor necrosis factor-alpha.
